# Impact of major hepatectomy on recurrence after resection of hepatocellular carcinoma at CNLC Ib stage: a propensity score matching study

**DOI:** 10.1097/JS9.0000000000001879

**Published:** 2024-06-24

**Authors:** Kunyuan Jiang, Jingfei Li, Zihao Liu, Miao Chen, Wei Cai, Lianxin Liu, Dalong Yin

**Affiliations:** a Department of General Surgery, Anhui Provincial Hospital, Anhui Medical University; b Department of Hepatobiliary surgery, The First Affiliated Hospital of University of Science and Technology of China; c Department of Hepatobiliary Surgery and Centre for Leading Medicine and Advanced Technologies of IHM, The First Affiliated HospitalDivision of Life Sciences and Medicine, University of Science and Technology of China; d Anhui Province Key Laboratory of Hepatopancreatobiliary Surgery; e Anhui Provincial Clinical Research Center for Hepatobiliary Diseases, Hefei, Anhui, People’s Republic of China

**Keywords:** hepatectomy, hepatocellular carcinoma, propensity score matching, recurrence-free survival

## Abstract

**Objective:**

Patients with hepatocellular carcinoma (HCC) who undergo curative hepatectomy may experience varying remnant liver volumes. Our study aimed to evaluate whether the extent of liver resection has an effect on postoperative recurrence in HCC patients at China Liver Cancer Staging (CNLC) Ib stage.

**Methods:**

A retrospective analysis was conducted on 197 patients who underwent hepatectomy for a solitary HCC lesion measuring ≥5 cm (CNLC Ιb stage) between January 2019 and June 2022. Patients were divided into a major hepatectomy (MAH) group (*n*=70) and a minor hepatectomy (MIH) group (*n*=127) based on the extent of liver resection. Recurrence-free survival (RFS) was compared between the two groups. Propensity score matching (PSM) was employed to minimize bias in the retrospective analysis.

**Results:**

Patients who underwent MAH had a greater total complication rate than those who underwent MIH (35.7 vs. 11.8%, *P*<0.001). The median RFS was 14.6 months (95% CI: 11.1–18.1) for the MAH group and 24.1 months (95% CI: 21.2–27.1) for the MIH group (*P*<0.001). After PSM, patients who underwent MAH still had a greater total complication rate than those who underwent MIH (36.7 vs. 16.3%, *P*=0.037). The median RFS was 13.2 months (95% CI: 15.1–21.7) for the MAH group and 22.3 months (95% CI: 18.1–26.5) for the MIH group (*P*=0.0013). The Cox regression model identified MAH as an independent poor predictor for HCC recurrence (hazard ratios of 1.826 and 2.062 before and after PSM, respectively; both *P*<0.05).

**Conclusion:**

MIH can be performed with fewer postoperative complications and contributes to improved RFS in patients with HCC at CNLC Ιb stage compared to MAH. Parenchyma-sparing resection should be considered the first choice for these HCCs.

## Graphical abstract

**Figure:**
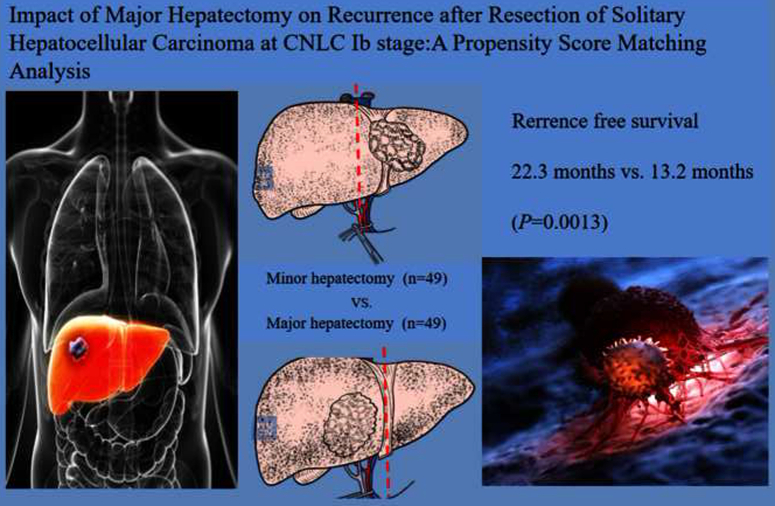

## Background

Highlights

- Minor hepatectomy significantly improved the recurrence-free survival (RFS) in China Liver Cancer Staging (CNLC) Ib hepatocellular carcinoma (HCC) patients.
- Major hepatectomy increased the risk of HCC recurrence by twice.
- Tumor size ≥10 cm was not an independent risk factor for RFS of HCC at CNLC Ib stage.
- Propensity score matching was employed to mitigate potential biases in this research.

Hepatocellular carcinoma (HCC) is the most common type of primary liver cancer and poses a significant health challenge globally^1^. The current curative methods for HCC include liver transplantation, ablation, and surgical resection, of which surgical resection is the most commonly used^2,3^. However, the high postoperative recurrence rate seriously affects the therapeutic effect of surgery, which is the most important factor for the long-term survival of patients after surgery^4,5^.

For a single HCC larger than 5 cm in diameter (CNLC Ib stage), due to its high degree of malignancy, major hepatectomy (MAH) is frequently recommended as the initial treatment option because of its theoretical advantage of ensuring wide cutting-edge and reducing postoperative recurrence rate^6^. However, this procedure reduces the remaining liver volume, which in turn raises the risk of postoperative complications such as liver failure^7,8^. On the contrary, while parenchyma-sparing resection may not provide the same assurance of a wide margin, it is considered a safer option as it aims to preserve as much liver parenchyma as possible. Recently, several studies have revealed that some surgery-related factors, such as ischemia-reperfusion injury, postoperative liver function, and remnant liver ischemia contribute to HCC recurrence^9–12^. These findings suggest that remnant liver function plays an important role in postoperative HCC recurrence. However, current research primarily focuses on short-term postoperative complications, with limited investigations into the long-term oncological outcomes of extensive hepatectomy.

Therefore, the objective of our study was to examine the clinical relevance between the extent of liver resection and HCC recurrence after hepatectomy. Considering extensive hepatectomy is often indicated for large HCCs, we specifically aimed at patients with HCCs measuring ≥5 cm (CNLC Ιb stage) to eliminate the confounding effect of tumor size.

## Methods

### Study design and patients

We retrospectively enrolled all patients who underwent radical surgery for HCC between January 2019 and June 2022. The inclusion criteria were as follows: (1) histologically confirmed solitary HCC with a size of ≥5 cm, (2) Eastern Cooperative Oncology Group score of 0 or 1, (3) preoperative indocyanine green retention rate at 15 min ≤15% and Child-Pugh A classification, and (4) no evidence of vascular invasion or extrahepatic disease. Patients who were not diagnosed with HCC or had an HCC measuring <5 cm, had confirmed tumor metastases or macrovascular invasion based on radiological examinations or died within 90 days after surgery were excluded. The enrolled patients were divided into two groups based on the extent of hepatectomy: a MAH group and a minor hepatectomy (MIH) group. MAH was defined as the removal of four or more Couinaud’s liver segments, while MIH was defined as the removal of three or fewer Couinaud’s segments^13^. The study protocol received approval from the Ethics Committee on 29 August 2023, and it was registered in the Clinical Trials.gov PRS (Protocol Registration and Results System). Our research has been reported in line with the strengthening the reporting of cohort, cross-sectional, and case–control studies in surgery (STROCSS) criteria^14^.

### Surgical procedure and definition

All patients underwent a comprehensive evaluation, including routine blood tests, liver function, and tumor marker tests. The selection of the type of resection was based on several factors, including the patient’s general condition, remnant liver volume, tumor location, and predicted surgical difficulty. If the remaining liver function was expected to be sufficient, as assessed by three-phase-enhanced computed tomography, MAH was considered.

Postoperative complications were assessed using the Clavien–Dindo classification system. In cases where a patient experienced multiple complications, only the complication with the highest grade was analyzed. Liver failure was defined based on the criterion of peak bilirubin levels exceeding 7 mg/dl^15^. The presence of ascites was determined by the presence of postoperative daily abdominal drainage exceeding 800 ml. Pleural effusion and peritoneal effusion were defined as fluid accumulation requiring puncture and drainage. Hypoproteinemia was defined as having a postoperative serum albumin of less than 30 g/l.

### Outcome endpoints and follow-up

The primary endpoint was recurrence-free survival (RFS). RFS was defined as the time from the time of radical surgery to the time of HCC recurrence. The secondary endpoint was postoperative complications related to liver function. The diagnostic criterion for recurrence was presence of recurrent lesions confirmed by at least one contrast-enhanced ultrasound, enhanced CT, or MRI scan of the abdomen. The final follow-up was conducted in August 2023.

### Statistical analysis

The baseline characteristics that may be related to recurrence, such as age, sex, BMI, HBsAg, AFP, liver cirrhosis, type of surgical procedure, intraoperative transfusion, surgical margin, tumor differentiation, tumor size, MVI, and whether transarterial chemoembolization (TACE) after surgery as adjuvant therapy were analyzed between the MAH and MIH groups. The 1:1 propensity score matching (PSM) method with a caliper of 0.1 was used to adjust the above variables between the two groups to overcome patient selection bias. Categorical variables were analyzed using *χ* ^2^ tests or 2-tailed Fisher exact tests. Kaplan–Meier method was used to analyze survival data, which were compared by the log-rank test. The prognostic factors mentioned above that may be linked to survival were comprehensively included in the Cox proportional hazards model. Variables with a *P-*value less than 0.05 in univariate analyses were included in the multivariate analyses to determine the adjusted hazard ratios (HRs) and 95% CIs. *P*-values <0.05 were considered to indicate statistical significance. All analyses were performed using statistical software (SPSS for Windows, version 26.0)

## Results

### Baseline characteristics

Between January 2019 and June 2022, a total of 203 patients with single HCCs measuring ≥5 cm were identified. After excluding six patients who died within 90 days (five in the MAH group and one in the MIH group), a final sample of 197 patients was included in this study. Among these patients, 70 underwent MAH (including 62 right hepatectomies, 4 extended right hepatectomies, and 4 extended left hepatectomies), while 127 patients underwent MIH (including 32 left hepatectomies, 10 right anterior hepatectomies, 17 right posterior hepatectomies, 11 mesohepatectomies, 20 left lateral hepatectomies, 21 right partial hepatectomies, 3 left partial hepatectomies, and 13 single segment hepatectomies). Using PSM analysis, 49 pairs of matched patients were identified from each group to compare outcomes.

Before PSM, patients in the MAH group were significantly younger and had larger HCC than patients in the MIH group (both *P*<0.05). Additionally, the MAH group exhibited higher levels of AFP, lower levels of ALB, and a greater proportion of intraoperative transfusion and positive MVI (all *P*<0.05). However, after PSM, there were no significant differences in any of the aforementioned characteristics between the two groups (*P*>0.05) (Table 1).

**Table 1:** Baseline demographic and disease characteristics before and after PSM.

	Before PSM			After PSM		
Patients	MAH (*n*=70)	MIH (*n*=127)	*P*	MAH (*n*=49)	MIH (*n*=49)	*P*
Age≥ 65years, n(%)	13 (18.6)	42 (33.1)	0.030	12 (24.5)	14 (28.6)	0.647
Male, *n* (%)	61 (87.1)	106 (83.5)	0.492	42 (85.7)	42 (85.7)	1.000
BMI ≥24, *n* (%)	27 (38.6)	58 (45.7)	0.336	20 (40.8)	13 (26.5)	0.135
Positive for HBsAg, *n* (%)	46 (65.7)	79 (62.2)	0.624	32 (65.3)	33 (67.3)	0.831
Albumin ≥40 g/l	23 (32.9)	66 (52.0)	0.010	20 (40.8)	22 (44.9)	0.683
AFP ≥400 ng/ml, *n* (%)	41 (58.6)	55 (43.3)	0.040	26 (53.1)	23 (46.9)	0.544
Liver cirrhosis, *n* (%)	39 (55.7)	79 (62.2)	0.374	29 (59.2)	27 (55.1)	0.683
Laparoscopical resection, *n* (%)	17 (24.3)	49 (38.6)	0.042	14 (28.6)	16 (32.7)	0.661
Intraoperative transfusion, *n* (%)	26 (37.1)	30 (23.6)	0.044	17 (34.7)	12 (24.5)	0.269
Surgical margin >1 cm, *n* (%)	27 (32.6)	41 (32.3)	0.374	14 (28.6)	16 (32.7)	0.661
Tumor size >10 cm, *n* (%)	37 (52.9)	27 (21.3)	<0.001	17 (34.7)	18 (36.7)	0.833
Poor differentiation, *n* (%)	31 (44.3)	42 (33.1)	0.119	19 (38.8)	15 (30.6)	0.664
Positive for MVI, *n* (%)	64 (91.4)	94 (74.0)	0.003	43 (87.8)	42 (85.7)	0.766
Adjuvant TACE after surgery, *n* (%)	22 (31.4)	42 (33.1)	0.814	17 (34.1)	11 (22.4)	0.180

AFP, alpha-fetoprotein; MVI, microvascular invasion; TACE, transarterial chemoembolization.

### Postoperative complications and recurrence patterns

Postoperative outcomes are shown in Tables 2, 3. Before PSM, the total morbidity was 35.7% in the MAH group and 11.8% in the MIH group (*P*<0.001). The types of complications according to Clavien–Dindo grades II and III differed between the two groups (both *P*<0.05). After PSM, the total morbidity was 36.7% in the MAH group and 16.3% in the MIH group (*P*=0.037). The types of complications, according to Clavien–Dindo grade, did not differ between the two groups.

**Table 2:** Postoperative outcomes before and after PSM.

	Before PSM			After PSM		
	MAH (*n*=70)	MIH (*n*=127)	*P*	MAH (*n*=49)	MIH (*n*=49)	*P*
Complications related to liver function, *n*%	25 (35.7)	15 (11.8)	<0.001	17 (36.7)	8 (16.3)	0.037
Clavien–Dindo grade						
II	12 (17.1)	10 (7.9)	0.048	8 (16.3)	6 (12.2)	0.564
Ascites	1	1		1	0	
Biliary leakage	0	7		0	5	
Hypoproteinemia	11	2		7	1	
III	8 (11.4)	3 (2.4)	0.020	6 (12.2)	1 (2.0)	0.050
Peritoneal effusion	7	1		5	0	
Pleural effusion	1	2		1	1	
IV	5 (7.1)	2 (1.6)	0.106	3 (6.1)	1 (2.0)	0.362
Liver failure	5	2		3	1	
Recurrence patterns						
Total recurrences	51 (72.9)	60 (47.2)	<0.001	37 (75.5)	24 (49.0)	0.007
Intrahepatic recurrence	27 (38.6)	43 (33.9)	0.508	22 (44.9)	14 (28.6)	0.094
Solitary	9 (12.9)	24 (18.9)	0.277	8 (16.3)	7 (14.3)	0.779
Multiple	18 (25.7)	19 (15.0)	0.064	14 (28.6)	7 (14.3)	0.085
Extrahepatic recurrence	24 (34.3)	17 (13.4)	0.001	15 (30.6)	10 (20.4)	0.247

MAH, major hepatectomy; MIH, major hepatectomy.

**Table 3:** Univariate and multivariate analysis for RFS in patients with HCC ≥5 cm Undergoing MAH/MIH.

		Univariate analysis			Multivariate analysis		
RFS	Number	HR	CI	*P*	HR	CI	*P*
All patients (*n*=197)							
Age (<65/≥ 65 years)	142/55	0.840	0.547–1.290	0.425			
Sex (female/male)	30/167	0.738	0.421–1.293	0.288			
BMI (>24/≥24)	112/85	1.398	0.961–2.035	0.080			
HBsAg (negative/positive)	72/125	1.183	0.796–1.758	0.405			
ALB (<40/≥40 g/l)	108/89	0.808	0.553–1.180	0.269			
AFP (<400/≥400 ng/ml)	101/96	1.492	1.024–2.175	0.037	1.290	0.882–1.887	0.190
Liver cirrhosis (negative/positive)	79/118	1.296	0.876–1.916	0.194			
Laparoscopical resection (no/yes)	131/66	0.771	0.512–1.159	0.211			
Intraoperative transfusion (no/yes)	141/56	1.361	0.914–2.029	0.129			
Surgical margin (<1/≥1 cm)	129/68	0.956	0.643–1.421	0.824			
Tumor size (5–10/≥10 cm)	120/77	1.586	1.082–2.324	0.018	1.073	0.702–1.641	0.745
Poor differentiation (no/yes)	124/73	1.118	0.759–1.646	0.573			
MVI (negative/positive)	39/158	2.497	1.370–4.552	0.003	1.949	1.042–3.647	0.037
Adjuvant TACE after surgery (no/yes)	133/64	1.362	0.926–2.002	0.116			
MIH/MAH	127/70	2.150	1.475–3.135	<0.001	1.826	1.206–2.765	0.004
Patients after PSM (*n*=98)							
Age (<65/≥ 65 years)	72/26	1.118	0.639–1.958	0.695			
Sex (female/male)	14/84	0.965	0.459–2.031	0.926			
BMI (>24/≥24)	65/33	1.757	1.054–2.929	0.031	1.545	1.217–3.494	0.100
HBsAg (negative/positive)	33/65	1.239	0.720–2.134	0.439			
Albumin (<40/≥40 g/l)	56/42	0.994	0.599–1.648	0.980			
AFP (<400/≥400 ng/ml)	49/49	1.388	0.838–2.299	0.202			
Liver cirrhosis (negative/positive)	42/56	1.612	0.950–2.736	0.077			
Laparoscopical resection (no/yes)	68/30	0.596	0.332–1.069	0.083			
Intraoperative transfusion (no/yes)	69/29	1.544	0.914–2.607	0.104			
Surgical margin (<1/≥1 cm)	68/30	1.140	0.662–1.964	0.636			
Tumor size (5–10/≥10 cm)	63/35	1.329	0.797–2.217	0.276			
Poor differentiation (no/yes)	64/34	1.106	0.595–1.735	0.954			
MVI (negative/positive)	13/85	1.538	0.661–3.578	0.317			
Adjuvant TACE after surgery (no/yes)	70/28	1.644	0.973–0.777	0.063			
MIH/MAH	49/49	2.219	1.319–3.731	0.003	2.062	1.217–3.494	0.007

AFP, alpha-fetoprotein; MAH, major hepatectomy; MIH, major hepatectomy; MVI, microvascular invasion; TACE, transarterial chemoembolization.

During the entire follow-up, 72.9% (51/70) patients in the MAH group and 47.2% (60/127) patients in the MIH group experienced postoperative recurrence (*P*<0.001). Patients in the MAH group had a greater incidence of extrahepatic recurrences than those in the MIH group (34.3 vs. 13.4%, *P*=0.001). The incidence of intrahepatic recurrence was similar between the two groups. After PSM, 37 patients in the MAH group and 24 patients in the MIH group developed postoperative recurrence (*P*=0.007). Furthermore, there were no significant differences observed in the types of recurrences between the two groups (both *P*>0.05) (Table 2).

### Survival analysis before and after PSM

**Figure 1:**
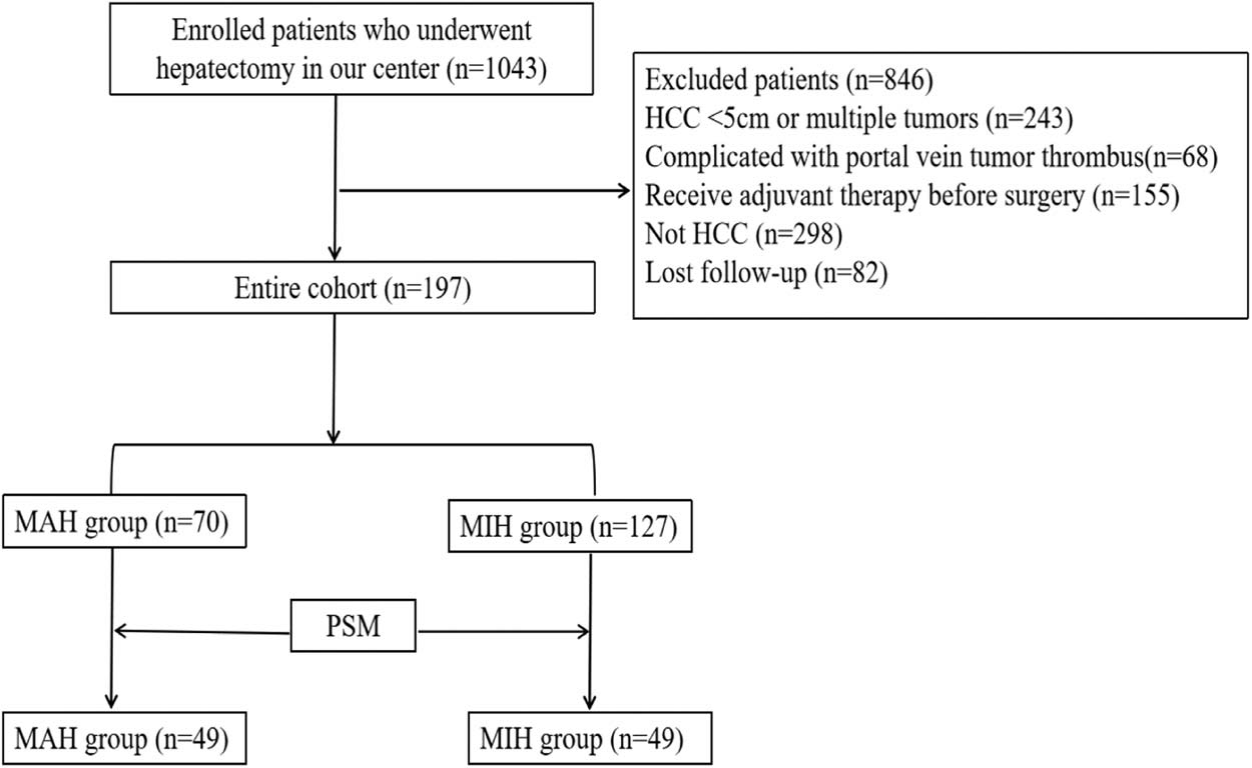
Flowchart of this study.

The median follow-up time was 24 months. Survival analysis between the two groups revealed significant differences in terms of RFS. The median RFS was 14.6 months (95% CI: 11.1–18.1) for MAH group and 24.1 months (95% CI: 21.2–27.1) for MIH group (*P*<0.001). (Fig. 1A). After PSM, patients in the MAH group continued to exhibit significantly poorer RFS than those in the MIH group. The median RFS was 13.2 months (95% CI: 15.1–21.7) for MAH group and 22.3 months (95% CI: 18.1–26.5) for MIH group (*P*=0.0013) (Fig. 1B).

According to the Cox regression model, univariate analysis of all patients revealed that AFP ≥400 ng/ml, tumor size >10 cm, positive MVI and MAH were associated with an increased risk of recurrence. Multivariate analysis identified two independent predictors of HCC recurrence: MVI positive (HR, 1.949; 95% CI: 1.042–3.647; *P*=0.037) and MAH (HR, 1.826; 95% CI: 1.206–2.765; *P*=0.004). When analyzing recurrence in patients selected through the propensity model, both univariate and multivariate analyses indicated that MAH remained the only significant predictor for poor RFS (HR, 2.062; 95% CI: 1.217–2.494; *P*=0.007).

## Discussion

Major hepatic resections are increasingly being performed for large HCCs to achieve complete removal and negative margin. However, the high incidence of postoperative recurrence remains a significant challenge even if R0 resection is achieved^16^. Currently, the main concern associated with MAH is the occurrence of postoperative complications, such as liver failure due to inadequate remnant liver function^17,18^. According to recent studies, MAH itself may have an adverse effect on HCC recurrence due to surgery-related liver damage. Thus, there is a need to further discuss the impact of MAH on long-term surgical outcomes. In this study, we chose a cutoff of the removal of four Couinaud’s liver segments to divide patients with HCCs measuring ≥5 cm into two subgroups to further investigate the impact of remnant liver volume on postoperative prognosis.

During the initial analysis of unselected patients, the MAH group was found to be younger and to have greater tumor burdens, resulting in lower ALB levels, higher AFP levels, fewer laparoscopic resections, more intraoperative transfusions, and a greater incidence of MVI positivity. These differences may have a confounding bias in the endpoint outcomes of our study. However, after 1:1 PSM, both matched groups achieved comparable baseline characteristics. It is not surprising that those who underwent MAH experienced more postoperative complications due to less remnant liver volume. Overall, both groups were successfully discharged following their operations. This suggests that with advancements in surgical technology, MAH can be performed safely, even for patients with liver cirrhosis.

**Figure 2:**
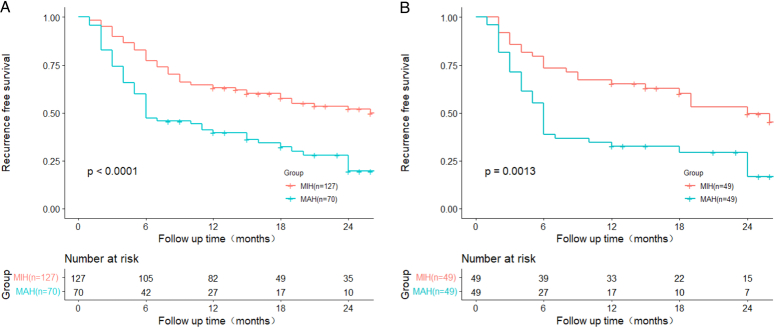
Recurrence-free survival before (A, *P*<0.0001) and after (B, *P*=0.0013) propensity score matching for patients who underwent major hepatectomy versus minor hepatectomy for hepatocellular carcinoma measuring ≥5 cm.

**Figure 3:**
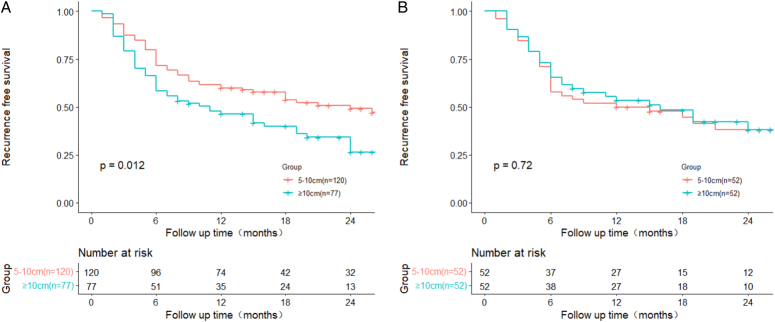
Recurrence-free survival before (A, *P=*0.012) and after (B, *P*=0.72) propensity score matching for patients with hepatocellular carcinoma measuring 5–10 cm versus those with HCC measuring ≥10 cm.

MVI has been widely reported to be the risk factor with the strongest association with recurrence in recent years^19,20^. In this study, the Cox multivariate model identified MVI as a negative predictor of RFS in unselected patients, which is consistent with previous conclusions. However, this conclusion was not observed after PSM, possibly due to the smaller number of MVI-negative patients, which leads to a lack of testing ability. Tumor size is an another important factor affecting postoperative recurrence. Most studies defined tumor size >5 cm as a risk factor for HCC recurrence^21–23^. Generally, a larger tumors often indicates a greater degree of malignancy and a worse prognosis^24,25^. However, when we further grouped the HCC with a diameter larger than 5 cm and bounded by 10 cm, it was found that a diameter exceeding 10 cm was not identified as an independent risk factor impacting prognosis. To make this conclusion more reliable, we used similar statistical methods to compare the prognosis of 5–10 cm HCCs with that of HCCs ≥10 cm, and the two groups had similar postoperative survival outcomes (Figs 2, 3). This conclusion reflected the complexity of assessing prognostic factors when evaluating the impact on patients’ outcomes. And it is suggested that factors other than tumor diameter may play a primary role in influencing the prognosis of patients with HCC larger than 5 cm.

Recent studies have highlighted the potential risk of liver resection itself as an independent factor for HCC recurrence due to impaired liver function caused by surgical damage. Additionally, the degree of underlying liver injury, such as background liver cirrhosis, has been shown to correlate with surgical outcomes in HCC^26^. These all emphasized the critical role of postoperative residual liver function in influencing the outcomes of surgical interventions for HCC^23,27^. It can also explain a recent interesting conclusion that patients with early-stage HCC seem to benefit more from anatomical hepatectomy^28–31^. Standard anatomical resection may result in a smaller remnant liver volume and posthepatectomy liver dysfunction, especially in patients with impaired liver background, such as cirrhosis or large HCC. This, in turn, can affect the outcome of anatomical hepatectomy. This assumption was also supported by another study that indicated the choice between anatomic or parenchyma-sparing resection did not significantly impact survival outcomes, while tumor characteristics and underlying liver function were significant determinants^11^.

In this study, we hypothesize that the extent of resection, as the most relevant indicator of remnant liver function^32^, could be attributed to postoperative recurrence, and the results confirmed this hypothesis. Based on our findings, the MAH group had poorer RFS than the MIH group. The Cox multivariate model identified MAH as the only negative predictor of RFS both before and after PSM.

Our findings might have an effect on clinical practice. MAH is a significant determinant of poor prognosis for patients with solitary HCC measuring ≥5 cm, a parenchymal-sparing approach should be considered the first choice for these patients^33,34^. If MAH is unavoidable, certain types of preoperative treatments, such as portal vein embolization or conversion therapy, which allows for an increase in liver volume, or neoadjuvant therapy to downstage the HCC tumor, should be considered to improve prognosis^35,36^. Interventional therapy has also shown promising results in the treatment of large unresectable HCC and is expected to become an alternative treatment option to surgical resection^37,38^.

The limitations of this study include its retrospective nature and relatively short follow-up duration. Even with careful PSM analysis, selection bias may not have been completely avoided. Moreover, considering that treatments after recurrence such as reresection, microwave ablation, TACE, and palliative treatment differed between the two groups, another important endpoint overall survival was not included in this study. Further studies with well-designed, long-term follow-up series and large-scale multicenter sample sizes are needed to validate our findings.

## Conclusion

Based on our results, compared to MAH, MIH was associated with fewer complications and better oncologic results when properly conducted. Therefore, considering the extent of liver resection when evaluating the prognosis of patients undergoing hepatectomy is essential. By preserving sufficient remnant liver volume, surgeons can potentially improve postoperative liver function, reduce complications, and enhance long-term oncological outcomes. Extensive hepatectomy, even if it can be performed safely, should be chosen with caution in view of the poor prognosis.

## Ethical approval

The study protocol was approved by the Ethics Committee of the First Affiliated Hospital of the University of Science and Technology of China (ID:2023-RE-285).

## Consent

Written informed consent for participation was not required for this study in accordance with the national legislation and the institutional requirements.

## Source of funding

This work was supported by the National Natural Science Foundation of China (No. 82172071).

## Author contribution

K.J.: study design, writing – original draft, and writing – review; J.L.: study design, data acquisition, and analysis; Z.L.: data acquisition and analysis; M.C.: methodology and interpretation of data; W.C.: methodology, software, and validation; L.L.: review and revise it critically for important intellectual content; D.Y.: guarantor of the article, project administration, funding acquisition, review, and edit.

## Conflicts of interest disclosure

The authors declare that the research was conducted in the absence of any commercial or financial relationships that could be construed as a potential conflict of interest.

## Research registration unique identifying number (UIN)

NCT 06390228.

## Guarantor

Dalong Yin, Ph.D, Hefei, Anhui, 230001, People’s Republic of China. Tel.: +86 18110984879. E-mail: doctoryin@ustc.edu.cn; Lianxin Liu, Ph.D, MD, Hefei, Anhui 230001, People’s Republic of China. Tel.: +86 13845159888. E-mail: liulx@ustc.edu.cn; Wei Cai, Ph.D, Hefei, Anhui 230001, People’s Republic of China. Tel.:+86 15625067917. E-mail: dr_caiwei@yeah.net.

## Data availability statement

The datasets used and analyzed during the current study are available from the corresponding author upon reasonable request.

## Provenance and peer review

Not applicable.
